# Evaluation of the Efficacy of an Artificial Intelligence-Based Assessment and Correction System in the Rehabilitation of Patients Following Anterior Cruciate Ligament Reconstruction Surgery

**DOI:** 10.3390/jcm15020575

**Published:** 2026-01-10

**Authors:** Tingting Zhu, Ying Huang, Jingjing Pu, Chaolong Wang, Min Ruan, Ping Lu, Xiaojiang Yang, Nirong Bao, Yueying Chen, Aiqin Zhang

**Affiliations:** 1 Department of Orthopedics, Jinling Clinical Medical College, Nanjing Medical University, Nanjing 210002, China; zhutingting2025@126.com; 2 Department of Orthopedics, Nanjing Jinling Hospital, Affiliated Hospital of Medical School, Nanjing University, Nanjing 210002, China; 13915909092@163.com (Y.H.); 18251830887@163.com (J.P.); wangchaolong0130@163.com (C.W.); 17798534025@163.com (M.R.); csjunjie@163.com (P.L.); xiaojiangyang@163.com (X.Y.); bnrbnr@sina.com (N.B.); 3 Department of Medical Information Data Room, Jinling Clinical Medical College, Nanjing Medical University, Nanjing 210002, China

**Keywords:** artificial intelligence, anterior cruciate ligament, rehabilitation, functional, evaluation, functional

## Abstract

**Background:** Arthroscopic anterior cruciate ligament (ACL) reconstruction is widely recognised as the primary treatment for ACL injuries. However, with the increasing incidence of sports-related injuries and growing demand for rehabilitation services, conventional rehabilitation models—largely reliant on therapists’ experience and subjective assessment—are increasingly insufficient to meet the clinical need for precise and individualised rehabilitation programmes. This study aimed to evaluate the effectiveness of a rehabilitation protocol incorporating an artificial intelligence (AI)-based assessment and correction system on functional recovery following ACL reconstruction. **Methods:** Using convenience sampling, 80 patients undergoing ACL reconstruction between June to December 2024 were recruited for this randomised controlled trial. Participants were randomly assigned to either a control group (*n* = 40), which received conventional functional exercise training, or a trial group (*n* = 40), which received rehabilitation intervention guided by an AI-based assessment and correction system. Knee function scores (Lysholm score, IKDC score), Berg Balance Scale (BBS) scores, joint range of motion (ROM), and rehabilitation exercise compliance scores were collected and analysed 1, 2, 3, and 4 months postoperatively. **Results:** Compared with the control group, the trial group demonstrated significantly greater improvements in Lysholm score, IKDC score, BBS score, and active knee joint ROM (*p* < 0.05) at postoperative assessment points. Additionally, rehabilitation exercise adherence was significantly higher in the trial group compared to the control group (*p* < 0.05). **Conclusions:** Rehabilitation protocols integrating AI-based assessment and correction systems effectively enhance knee function recovery, joint mobility and balance ability following ACL reconstruction. Moreover, these protocols significantly improve rehabilitation exercise adherence, demonstrating superior efficacy compared to conventional rehabilitation approaches. This digital rehabilitation model represents an efficient and promising intervention for postoperative ACL rehabilitation.

## 1. Introduction

Arthroscopic anterior cruciate ligament (ACL) reconstruction is currently regarded as the standard treatment injuries, ref. [1] primarily aiming to restore knee stability and prevent secondary damage to the meniscus and articular cartilage [2,3]. Postoperative rehabilitation following ACL reconstruction is an essential component in restoring knee stability and functional mobility [4]. However, with the rising prevalence of sports injuries and increasing rehabilitation demands, traditional rehabilitation models have revealed notable limitations.

Conventional rehabilitation largely depends on therapists’ experiential judgement and subjective evaluation, which may result in inconsistent assessment standards, insufficient quantitative monitoring, and delayed feedback. Consequently, they can no longer adequately meet the urgent clinical need for developing precise, personalised rehabilitation programmes. Previous research indicates that exercise adherence among ACL reconstruction patients is suboptimal [5]. Inadequate adherence is associated with delayed functional recovery, reduced quality of life, and waste of healthcare resources, directly impacting postoperative rehabilitation outcomes [6,7]. In recent years, advances in artificial intelligence (AI) and biomechanical analysis have facilitated rehabilitation exercises for patients with ACL reconstruction, offering new opportunities to enhance rehabilitation effectiveness and patient engagement [8].

The present study focuses on the application of ‘Intelligent Functional Movement and Physical Fitness Assessment System (ZD-200S-JG)’ and the ‘Intelligent Movement Analysis and Correction System (ZD-200S-ZXJ)’. These systems employ non-wearable three-dimensional motion capture to acquire the precise kinematic data without the need for body-mounted markers, enabling the construction of a digital human model for objective functional assessment. We hypothesised that this AI-based system could improve rehabilitation adherence and functional outcomes through three key mechanisms: (1) objective and quantitative assessment with visualised feedback to enhance patients’ understanding of recovery progress; (2) generation of individualised exercise prescriptions combined with visual guidance to ensure safe and effective training; and (3) real-time motion correction and streamlined training processes to improve training efficiency and motivation.

This pilot randomised controlled trial aimed to investigate the application advantages of an AI-based assessment and correction system in the rehabilitation of patients following ACL reconstruction. The primary objective of this study is to explore the feasibility of the research design (including randomization procedures, outcome measurement methods, and patient adherence to the intervention). The study sought to establish a comprehensive digital rehabilitation model integrating precise assessment, personalised prescription, and visual guidance for follow-up training, thereby providing evidence to support standardised, efficient, and individualised rehabilitation pathways in clinical sports medicine.

## 2. Materials and Methods

### 2.1. Study Design and Participants

This randomised controlled trial employed a convenience sampling method, recruiting 80 patients who underwent ACL reconstruction surgery and were admitted to the Sports Medicine Ward of the Orthopaedic Department at the Eastern Theatre Command General Hospital between June and December 2024. Eligible patients were randomly assigned to either a control group or a trial group using a random number table.

Inclusion criteria: (1) Diagnosis of ACL rupture confirmed by magnetic resonance imaging (MRI); (2) arthroscopic single-bundle ACL reconstruction performed; (3) aged 18–50 years, gender unrestricted, with no restriction on sex; (4) patients fully informed of the study process and objectives, demonstrating understanding and voluntarily signing the informed consent form, willing to cooperate with rehabilitation programme.

Exclusion criteria: (1) Concurrent structural knee injuries including posterior cruciate ligament or collateral ligament injuries, fractures, or other lesions of the ipsilateral lower limb; (2) presence of severe organic diseases that would preclude participation in rehabilitation training.

Withdrawal criteria: (1) Patients experiencing clinical deterioration during the study period, rendering continued rehabilitation inappropriate; (2) patients voluntary withdrawing from the study.

This study was approved by the Ethics Committee of Nanjing Jinling Hospital (Approval No. 2023JLHGZRDWLS-00018) and conducted in accordance with the Declaration of Helsinki.

The sample size was calculated using a two-sample means comparison formula. Based on preliminary estimates, a sample size of 66 participants was required, with 33 cases per group. Allowing for a potential dropout rate of 20%, the final sample size was determined to be 40 participants per group. During the study period, one participant in the trial group withdrew approximately one month after enrolment due to job relocation. This participant had not initiated AI-based rehabilitation training and did not complete any follow-up assessments. The withdrawal was unrelated to the intervention. Ultimately, 79 patients completed all follow-up and were included in the statistical analysis. Comparisons of general baseline characteristics between the two groups revealed no statistically significant differences (*p* > 0.05), indicating comparability (Table 1).

### 2.2. Research Methodology

#### 2.2.1. Establishment of the Research Team

The multidisciplinary research team comprised one consultant physician, one head nurse, two orthopaedic rehabilitation therapists, one master’s student in nursing, and one senior nurse. Guided by the principle of integrated medical, nursing and rehabilitation care, the consultant surgeon and head nurse led the team in developing of the rehabilitation protocol based on the intelligent functional movement assessment and correction system.

The master of nursing candidate is responsible for retrieving and evaluating relevant domestic and international literature concerning post-operative functional rehabilitation for ACL reconstruction, assessing the feasibility of intervention, and participating in its implementation of the expanded sample intervention. Rehabilitation therapists were responsible for adjusting training content and intensity according to patients’ recovery status. Outcome assessments were conducted by rehabilitation therapists who were blinded to group allocation.

#### 2.2.2. Develop Rehabilitation Programmes Based on Intelligent Functional Movement and Physical Fitness Assessment Systems

Addressing the physiological characteristics of ACL reconstruction patients, such as potential balance dysfunction arising from weight-bearing restrictions on the affected limb post-surgery, the research team conducted a systematic reviewed relevant literature and ACL rehabilitation guidelines to gain an in-depth understanding of the application principles and methodologies underlying intelligent assessment and correction systems. Building on preliminary pilot studies with a small sample sizes, the rehabilitation protocol was further refined and optimised through interdisciplinary consultation with hospital specialists, engineers responsible AI-based assessment system, and rehabilitation therapists. This process led to the development of a scientific, systematic, and AI-driven rehabilitation programme integrating assessment and guided training-based rehabilitation programme for ACL reconstruction patients.

#### 2.2.3. Intervention Methods

##### Intervention Protocol for the Control Group

Participants in the control group received conventional rehabilitation training based on a standardised, time-phased rehabilitation protocol. The intervention relied primarily on the physiotherapist’s experience and clinical subjective assessment.

One week post-surgery:

① Ankle Pump Exercise: Perform slow, forceful ankle flexion and extension through the full range of motion. 20 reps/set, 5 sets/day.

② Quadriceps Isometric Contraction: Alternately contract and relax the anterior thigh muscles without inducing pain. 20 reps/set, 5 sets/day.

③ Hamstring isometric contractions: Place a rolled towel approximately 5 cm thick under the knee. Press the knee downward to contract the posterior thigh muscles, then relax. 20 reps/set, 5 sets/day.

④ Patellar Mobilisation: With the knee fully relaxed, gently glide the patella in all directions. Gradually increase the range of motion until it matches that of the unaffected side. 5 min/session, twice/day.

⑤ Weight-bearing Precautions: When getting out of bed, wear a brace to maintain knee extension. Ambulate with crutches and avoid weight-bearing on the affected limb.

⑥ Knee Extension Exercise: Elevate the heel on a towel approximately 20 cm high, allowing the knee to remain unsupported and suspended. Perform once in the morning and once in the afternoon, 20–30 min/session.

Two weeks post-surgery:

Continue all previous exercises and add:

Non-Weight-Bearing Knee Flexion Exercises: Perform controlled knee flexion without loading the limb. Adjust the brace to limit flexion to less than 60°, stopping at the onset of pain. 5 min/session, twice/day.

Four weeks post-surgery:

① Progressive Knee Flexion: Continue prior exercises and practice knee flexion while seated on the edge of a bed or stool. Gradually increase the flexion angle to 90°. 10 min/session, 2 sessions/day.

② Weight-Bearing Ambulation: Walk while wearing a brace adjusted to the maximum achievable flexion angle.

③ Resistance Training of the Affected Limb: With brace protection, shift body weight onto the unaffected leg while performing resistance exercises with the affected leg. Train in four directions. 15 reps/set, 4 sets/day.

Six weeks post-surgery

Continue all previous exercises. Knee flexion may progress beyond 100° as tolerated.

Eight–Sixteen weeks post-surgery

① Range of Motion Training: Continue all exercises. Knee flexion may progress to full range of motion.

② Heel Raises: If lower-limb stability is insufficient, use upper-limb support against a wall.

③ Step-Up Training: Step onto a stair using the affected leg as the leading leg. 10 reps/set, 4 sets/day.

During the perioperative period, diversified health education will be implemented alongside standardised pain management strategies, with a particular emphasis on strengthening perioperative education and rehabilitation guidance. The diversified education framework specifically includes the placement of QR codes in ward corridors to disseminate rehabilitation knowledge accompanied by continuously looped rehabilitation guidance videos and organising preoperative group-based educational sessions. Upon admission, patients will receive the ‘Anterior Cruciate Ligament Reconstruction Postoperative Rehabilitation Exercise Manual’, which comprehensively outlines preoperative preparations, step-by-step postoperative rehabilitation exercises, and essential precautions. This systematic, multi-tiered approach to education and communication is designed to enhance patients’ understanding of their diagnosis, treatment, and rehabilitation process. By reducing preoperative anxiety and postoperative uncertainty, it aims to improve treatment adherence, rehabilitation confidence, and active participation.

Following discharge, members of the research team who have undergone standardised training will conduct regular follow-up via WeChat and telephone. Home-based rehabilitation data will be collected through electronic questionnaires and patients will receive timely reminders and encouragement at key milestones throughout each phase of rehabilitation, guiding them to complete their prescribed rehabilitation exercises as schedule.

##### Intervention Protocol for the Trial Group

(1) Pre-intervention Preparation

① Standardised Training: Prior to intervention, the researcher conducted standardised training on rehabilitation protocols using the AI-based Assessment and Correction System for all participating patients and members of the research team. Relevant exercise programme materials were distributed to ensure consistency and understanding.

② Patient Assessment: Research team members conducted comprehensive evaluations of each patients’ physical condition, psychological status, comorbidities, exercise motivation, and feasibility of participation.

**Figure 1:**
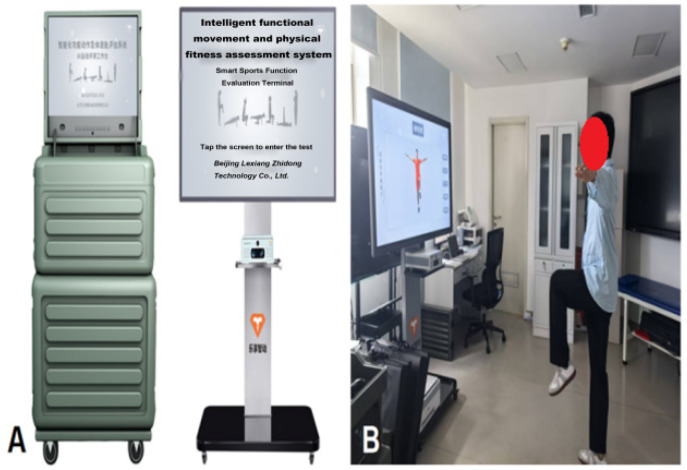
(**A**) Intelligent functional movement and physical fitness assessment system; (**B**) Patient undergoing movement evaluation.

**Figure 2:**
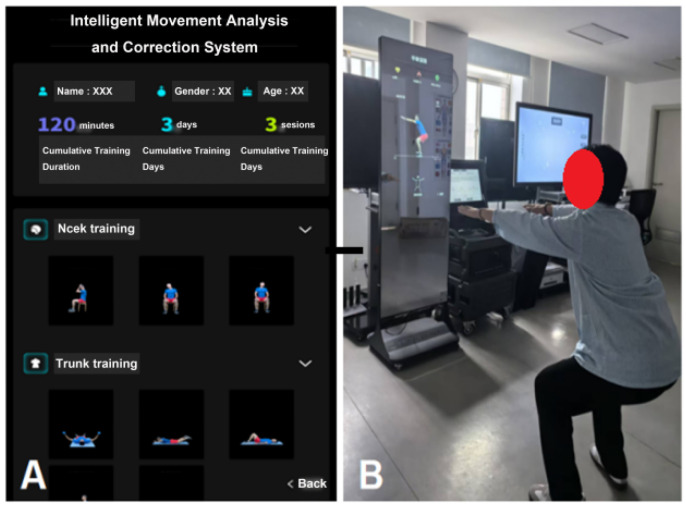
(**A**) Intelligent movement analysis and correction system interface display; (**B**) Patient following training instructions.

③ Environmental and Equipment Preparation: The Intelligent Functional Movement and Physical Fitness Assessment System (ZD-200S-JG) was employed for precise movement evaluation (Figure 1A,B); while the Intelligent Movement Analysis and Correction System (ZD-200S-ZXJ) was used to support exercise prescription monitoring and real-time movement correction (Figure 2A,B). All the above equipment was purchased from Lexiang Zhidong Technology Co., Ltd., Beijing, China. Prior to deployment, the AI-based motion assessment and training guidance system was fully debugged. The rehabilitation environment was prepared to ensure adequate lighting, suitable temperature, cleanliness, and the absence of obstructions.

④ Patient Preparation: Patients were instructed to wear loose-fitting clothing and well-fitting athletic shoes. All training components were completed under the supervision and guidance of rehabilitation therapists.

Pre-intervention Training and Knowledge Implementation

To ensure consistent understanding and operation of the intelligent rehabilitation system among trial participants and research team members, this study conducted systematic, tiered unified training prior to intervention, as detailed below:

① Training Content and Structure

The training process comprises two components: patient training and operator training. Patient training covers an introduction to the system’s fundamental principles and functions, demonstration of operational procedures, standards for movement execution and safety precautions, methods of device interaction, and education on rehabilitation adherence. This aims to help patients develop a proper understanding of digital rehabilitation and acquire the necessary skills for its use. Operator training focuses on system operation procedures, data management and interpretation, prescription fine-tuning principles, safety monitoring, and communication techniques. This ensures the research team can implement interventions in a standardised and secure manner.

② Training Methods and Duration

Training combines group instruction, video demonstrations, hands-on simulations, and individual guidance. Patient training lasts approximately 30 min, while operator training takes about 1 h. A brief assessment or Q&A session follows each training session to ensure trainees grasp key content.

③ Patient Cognitive Implementation Measures

To enhance patients’ understanding and acceptance of the intervention methods, we implemented a multi-modal cognitive implementation strategy: distributing illustrated versions of the “Smart Rehabilitation System User Guide”; screening system operation videos and facilitating simulated experiences; conducting interactive Q&A sessions post-training to promptly address patient inquiries; having patients sign informed consent forms after training to confirm comprehension of the research process and system usage; and maintaining continuous communication by rehabilitation therapists throughout the intervention to reinforce cognitive understanding and operational feedback.

(2) Implementation of the Intervention Programme

**Figure 3:**
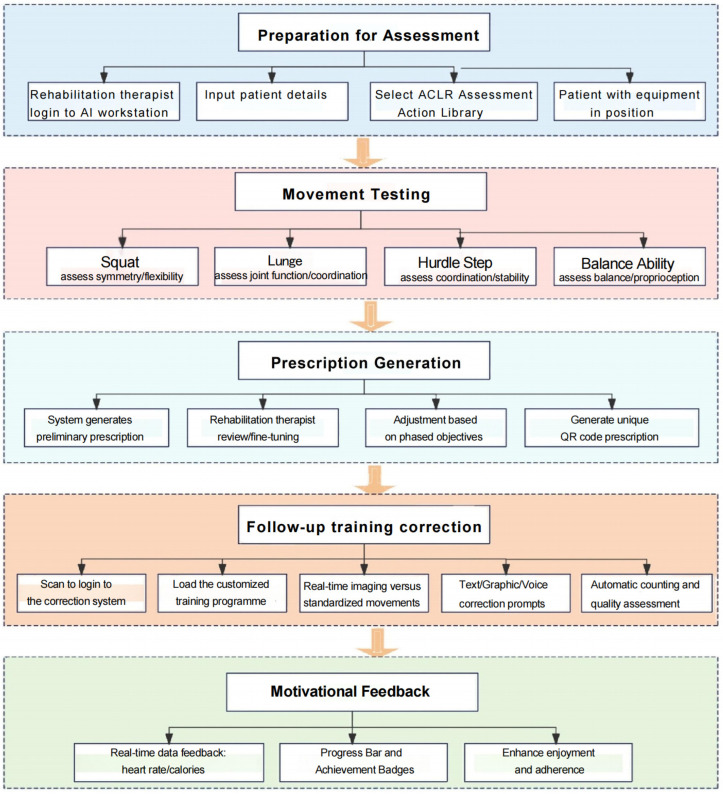
Flowchart of the rehabilitation programme for the AI-based assessment and correction system.

This programme utilised an ‘*Intelligent Functional Movement and Physical Fitness Assessment System*’ to evaluate functional movements, generate physical assessment reports and develop personalised exercise prescriptions. An ‘*Intelligent Movement Analysis and Correction System*’ was subsequently applied for follow-up training. The intervention comprises three phases, forming a closed-loop rehabilitation process of ‘assessment-prescription-training-feedback’ (Figure 3). The programme was delivered at the orthopaedic rehabilitation centre, commencing one month post-surgery with three sessions per week over a 12-week period. Each training session was supervised, guided, and documented by a rehabilitation therapist and a senior nurse to ensure both safety and effectiveness. The initial comprehensive functional assessment was scheduled around four weeks post-surgery, a timing aligned with current consensus and evidence on accelerated ACL reconstruction rehabilitation [4]. Research indicates [9] that at this stage, most patients can achieve ≥110 ° knee flexion under structured rehabilitation guidance, meeting the range of motion requirements for low-load closed-chain exercises and static balance testing. Furthermore, early standardised neuromuscular control training has been demonstrated to be both safe and beneficial [10], providing the theoretical and practical foundation for the AI-based movement assessment applied in this study.

① Warm-up (approximately 5–10 min): The rehabilitation therapist selected four dynamic stretching exercises from the AI motion analysis and correction system’s exercise library specifically designed for patients following ACL reconstruction (Table 2), Patients were guided through imitative training. This phase aimed to elevate heart rate, increase blood flow to target muscle groups, enhance muscle temperature and elasticity, prepare for subsequent precise assessment and high-quality training, and prevent exercise-related injury.

② Core Component: Precision Movement Assessment, Exercise Prescription Generation and AI-Guided Training (approximately 30–40 min). This phase represented the core of digital rehabilitation, fully leveraging the synergistic advantages of two intelligent systems (Table 3). Using non-wearable 3D motion capture technology, the system automatically generated quantitative physical assessment reports without requiring marker placement. The report included detailed biomechanical data such as movement scores, key joint ranges of motion, movement symmetry, stability ratings, and deviation angles. AI algorithms compared patient data with standard reference models to accurately identify specific functional deficits and impairments.

**Table 3:** Core Components of Training.

Phase	Steps	Content	Core Technology	Result	Personnel Role
A. Precision Movement Assessment	Login and Preparation	The rehabilitation therapist enters the patient’s basic information into the AI workstation and activates the ACLR-specific movement assessment module (comprising four designated movements). The patient, wearing close-fitting exercise attire and fitted with a smart heart rate armband, stands within the designated area.	Non-wearable three-dimensional motion capture technology, requiring no adhesive markers.	Test preparation completed; entering evaluation status.	Rehabilitation Therapist: Operating system, guiding the patient to complete standing position adjustments Patient: Completing test movements according to system voice and animated prompts.
	AI Motion Testing (Figure 4A–D)	Patients complete the following exercises following voice and animated prompts: 1. Squat: Assesses bilateral hip, knee, and ankle joint symmetry, flexibility, and stability; identifies abnormal movement patterns such as knee valgus. 2. Lunge: Evaluates dynamic stability, multi-joint coordination, and quadriceps flexibility. 3. Hurdle step: Assesses dynamic control in the sagittal and coronal planes, gait coordination, and core stability; detects potential pelvic compensation, insufficient knee stability, and ankle control deficits during stride. 4. Balance assessment: Single-leg stance (first unaffected side, then affected side) to quantify static balance and proprioception.	Three-dimensional motion capture and artificial intelligence real-time analysis technology	Generate a quantified physical fitness assessment report, including individual exercise scores, range of motion (ROM), symmetry, stability ratings, deviation angles, and other parameters.	
	Functional Impairment Identification	The system automatically compares patient movement data against standard models to accurately identify functional impairments, such as restricted flexion in the affected knee joint, diminished dynamic stability during squatting, and significant differences in bilateral balance capabilities.	AI-based algorithmic analysis	Identify primary functional impairments and rehabilitation priorities	
B. Personalised Exercise Prescription	Automatic Prescription Generation	Based on the assessment results, the system automatically generates an ACL reconstruction initial training programme with a high degree of safety. This programme includes training movements, sets, repetitions, target range of motion, and rest intervals.	AI prescription generation logic based on assessment data	Generate an initial personalised training prescription	Rehabilitation Therapist: Responsible for reviewing and adjusting prescriptions while retaining clinical decision-making authority. Patient: Provides subjective feedback on their experience.
	Manual adjustment of prescriptions	The rehabilitation therapist reviews and makes minor adjustments to the system-generated prescription based on the patient’s actual pain perception, fatigue levels, and phased rehabilitation objectives.	Integrating clinical experience with systematic recommendations.	Confirm final personalised prescription	
	Example of prescription content	The ACL reconstruction Prescription Library comprises the following representative exercises: Lateral raise squats, wide-stance slow squats, single-leg glute bridges, side-lying leg raises, seated knee tucks, five-time sit-to-stand test, forward lunges, leg swings, single-leg stance with eyes closed, static balance training, side lateral raises with rear toe taps, step test, three-metre shuttle walk, side-to-side shuffles, clap between legs, high knees with hands touching knees, etc. (Each prescription typically includes approximately six exercises, covering multidimensional training content such as muscle strength, joint range of motion, and balance function).	The prescribed exercise library encompasses multiple categories including static stretching, dynamic stretching, stability training, corrective training, and strength programmes. Lower-body training can be precisely targeted to specific muscle groups.	Generate a unique QR code for patients to scan and retrieve their personalised programme and training guidance.	
C.AI Follow-Up Training and Correction	Scanning to log in	Prior to the patient’s positioning at the Intelligent Motion Analysis and Correction System, scan the QR code to retrieve the personalised treatment plan. The system continuously monitors movement execution in real time, providing auditory or visual corrective prompts whenever deviations occur.	QR code technology	The system automatically loads the corresponding training programme.	Rehabilitation Therapist: Observe overall condition, ensure training safety, provide motivational support, deliver advanced technique guidance and health education. Patient: Engage in active training within an immersive interactive environment.
	Real-time feedback and correction	The system continuously monitors exercise execution in real time, providing auditory or visual corrective prompts when deviations occur (e.g., ‘Knees collapsing inward, please abduct them’ or ‘Insufficient squat depth’). It delivers immediate quality assessments for each movement (e.g., “Standard” or ‘Needs improvement’) and automatically tallies repetitions.	Real-time motion capture and analysis, image overlay comparison, artificial intelligence motion recognition and evaluation algorithms.	Ensure the quality of movements and the training volume are accurately completed.	
	Gamification and Incentives	The system displays real-time data including heart rate, calories burned, and duration, while enhancing engagement and adherence through elements such as progress bars and achievement badges.	Real-time biofeedback and gamification design.	Enhance patient engagement and adherence	

**Figure 4:**
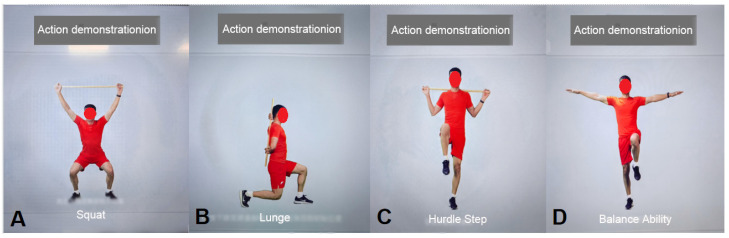
AI Movement Testing. (**A**) Squat; (**B**) Lunge; (**C**) Hurdle Step; (**D**) Balance Ability.

③ Stretching Exercises (approximately 5–10 min): Following the training session, patients perform static stretching exercises demonstrated by the AI motion correction system to relax and elongate the quadriceps, hamstrings, triceps surae, and gluteal muscle groups (Table 4). Each stretch position was held for 15–30 s. This phase aimed to enhance muscle and tendon flexibility, improve joint range of motion, alleviate muscle tension and soreness, and promote recovery. Finally, the senior nurse applied cryotherapy to the knee joint for 15 min to reduce potential post-exercise inflammatory responses.

(3) Quality Control of the Intervention Process

① Quality control of research subjects: Participants were recruited strictly in accordance with predefined inclusion and exclusion criteria. Subjects in different groups were managed in strict compliance with the study protocol to minimise confounding bias.

② Training of Intervention Personnel: All research team members underwent standardised training and competency assessment prior to the intervention. Data collection and recorded were performed exclusively by designated personnel. All records were carefully checked and verified to identify omissions or errors, which were corrected promptly. Any amendments required due to incorrect entries were documented, with the original error remaining visible and accompanied by the date and signature of the responsible researcher.

③ Compliance control: Throughout the study period, health education was strengthened to enhance participants’ health beliefs and adherence. Rehabilitation exercise techniques were demonstrated in detail, and patients and their families were instructed on relevant precautions. Clear guidance was provided for completing assessment scales, and ongoing communication was maintained to promptly address any issues arising during the research process.

#### 2.2.4. Assessment Criteria

① The Lysholm Knee Score is a widely used clinical instrument for evaluating clinical knee function, comprising eight components: limping, support, locking, instability, swelling, stair climbing, squatting, and pain. Total scores range from 0 to 100, with higher values indicating superior knee function and lower values indicating poorer function. Specifically, scores of 95–100 points are classified as excellent, 84–94 as good, 65–83 points as fair, and below 65 points as poor [11]. The overall Cronbach’s α coefficient for this scale is 0.68, demonstrating acceptable internal consistency as well as good convergent and discriminant validity [12].

② International Knee Documentation Committee Subjective Knee Evaluation Form (IKDC): The IKDC Subjective Knee Evaluation Form was first introduced in 1993 and has undergone several revisions since then. It comprises 18 items, including 10 items assessing knee function and 8 items evaluating ligament-related symptoms. The scale assesses knee pain, functional status, activities of daily living, and the ability to participate in sports, with total scores ranging from 0 to 100 [13]. Higher scores reflect better knee function. A study evaluating patients two years after anterior cruciate ligament (ACL) reconstruction demonstrated a significant correlation between IKDC scores and objective hop test results, indicating that the IKDC effectively reflects postoperative knee function in this population [14].

③ The Berg Balance Scale (BBS), developed by Katherine Berg in 1989, is a commonly used tool for assessing balance ability. It consists of 14 tasks, including standing up, sitting down, standing independently, standing with eyes closed, reaching forward with an outstretched arm, turning around, stepping up and down a step, and standing on one leg. Each item is scored on a 5-point scale from 0 to 4, yielding a maximum total score of 56. Scores of 0–20 indicate poor balance requiring wheelchair assistance; 21–40 indicate sufficient balance for safe ambulation with assistive devices such as a walker or cane; and 41–56 indicate good balance, allowing independent walking [15].

④ Active Range of Motion (ROM) of the Knee Joint: Active knee joint range of motion (ROM) was assessed before the intervention and at 4, 8, and 12 weeks post-intervention. Maximum knee flexion and extension angles were measured using a goniometer. During measurement, the centre of the goniometer was placed over the lateral femoral condyle, with the stationary arm aligned along the femoral midline toward the greater trochanter and the movable arm aligned with the fibular midline toward the lateral malleolus. The goniometer was secured to the affected limb while the patient actively flexed and extended the knee to the maximum tolerable range. Each measurement was performed three times, and the mean value was recorded as the knee joint ROM [16].

⑤ Functional exercise adherence. Assessed using the Orthopaedic Patient Functional Exercise Adherence Scale [10], comprising three dimensions: physical exercise adherence, psychological exercise adherence, and active learning exercise adherence, totalling 15 items. Each item employs a five-point Likert scale: Not at all possible, “Rarely possible, Occasionally possible, Mostly possible, Completely possible”, scored sequentially from 1 to 5 points, yielding a total score range of 15–75 points. Higher scores indicate better adherence. A total score ≤ 20 denotes low adherence, 21–54 indicates partial adherence, and ≥55 signifies high adherence. The Cronbach’s α coefficient for this scale is 0.930.

#### 2.2.5. Statistical Methods

Statistical analyses were performed using SPSS software (version 27.0, SPSS Inc., Chicago, IL, USA), with data double-entered, cross-checked, collated, and analysed. Continuous variables were expressed as mean ± standard deviation and compared using independent-samples *t*-tests. Categorical variables were analysed using chi-square tests. Repeated-measures analysis of variance was applied to compare outcomes between groups over time. All statistical analyses employed two-tailed tests, with *p* < 0.05 indicating statistically significant differences.

#### 2.2.6. AI Statement

The authors declare that artificial intelligence tools were used solely for language editing and stylistic improvement of the manuscript. The use of AI did not involve the generation, analysis, or interpretation of data, nor did it influence the study design, results, or scientific conclusions. All content was reviewed and approved by the authors, who take full responsibility for the integrity and accuracy of the work.

## 3. Results

### 3.1. Comparison of Knee Function and Balance Ability (Table 5, Table 6 and Table 7, Figure 5A–C)

Baseline Lysholm scores, International Knee Documentation Committee (IKDC) scores, and Berg Balance Scale (BBS) scores did not differ significantly between the control and trial groups (all, *p* > 0.05), indicating comparability at study entry. Following intervention, both groups demonstrated significant improvements in Lysholm scores, IKDC scores, and BBS scores at all time points compared with pre-intervention levels. The trial group consistently achieved higher scores than the control group at each time point, with statistically significant differences (*p* < 0.05).

**Table 5:** Lysholm scores ($\bar{X}$ ± s).

Group	One Month Post-Surgery	Two Months Post-Surgery	Three Months Post-Surgery	Four Months Post-Surgery
Control Group	47.83 ± 4.92	54.95 ± 5.39	60.65 ± 5.11	66.08 ± 5.08
Trial Group	47.49 ± 4.52	57.51 ± 4.73	64.10 ± 4.85	68.54 ± 4.54
*t*	0.32	2.25	3.08	2.27
*p*	0.7516	0.0276 *	0.0029 **	0.0260 *

Note: * indicates significant difference (*p* < 0.05); ** indicates significant difference (*p* < 0.01).

**Table 6:** IKDC scores ($\bar{X}$ ± s).

Group	One Month Post-Surgery	Two Months Post-Surgery	Three Months Post-Surgery	Four Months Post-Surgery
Control Group	47.88 ± 4.08	54.08 ± 4.52	60.75 ± 5.45	65.15 ± 4.02
Trial Group	48.33 ± 4.91	57.18 ± 5.49	63.51 ± 3.89	68.00 ± 3.81
*t*	0.45	2.75	2.59	3.23
*p*	0.6529	0.0075 **	0.0115 *	0.0018 **

Note: * indicates significant difference (*p* < 0.05); ** indicates significant difference (*p* < 0.01).

**Table 7:** Berg Balance Scale scores ($\bar{X}$ ± s).

Group	One Month Post-Surgery	Two Months Post-Surgery	Three Months Post-Surgery	Four Months Post-Surgery
Control Group	34.00 ± 2.58	38.50 ± 1.13	41.70 ± 2.23	45.98 ± 3.12
Trial Group	33.95 ± 2.70	39.95 ± 1.96	43.49 ± 2.25	48.08 ± 2.26
*t*	0.09	4.04	3.55	3.39
*p*	0.9314	0.0001 ***	0.0007 ***	0.0011 **

Note: ** indicates significant difference (*p* < 0.01); *** indicates significant difference (*p* < 0.001).

**Figure 5:**
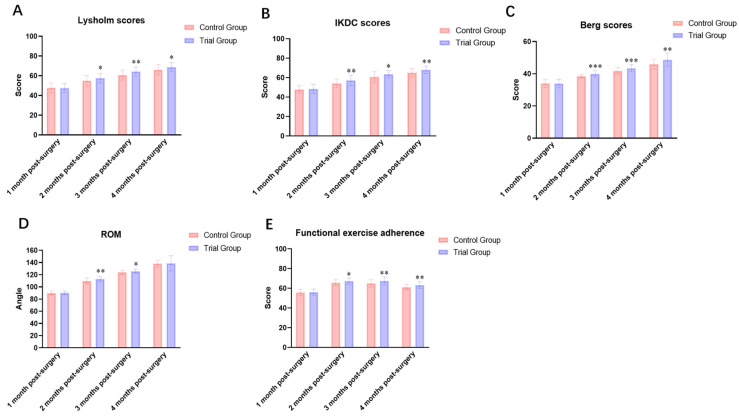
Intra-group and inter-group comparisons of evaluation indicators before and after intervention. (**A**) Lysholm score; (**B**) IKDC score; (**C**) Berg Balance Scale score; (**D**) Knee range of motion (ROM); (**E**) Functional exercise compliance score. Note: * indicates significant difference (*p*<0.05); ** indicates significant difference (*p* < 0.01); *** indicates significant difference (*p* < 0.001).

### 3.2. Comparison of Knee Joint Range of Motion (ROM) (Table 8 and Figure 5D)

Prior to intervention, no statistically significant difference were observed in knee joint ROM between the two groups (*p* > 0.05). Post-intervention, the trial group exhibited a sustained increase in knee ROM over time. Post-intervention analyses showed that knee joint range of motion increased progressively in both groups over time. However, the trial group demonstrated significantly greater improvements compared with the control group at postoperative months 2, 3, and 4 (all *p* < 0.05). The between-group differences became more pronounced with increasing rehabilitation duration.

**Table 8:** Knee Joint Range of Motion (ROM) ($\bar{X}$ ± s).

Group	One Month Post-Surgery	Two Months Post-Surgery	Three Months Post-Surgery	Four Months Post-Surgery
Control Group	90.00 ± 3.26	109.80 ± 4.87	123.90 ± 3.69	138.20 ± 5.87
Trial Group	90.15 ± 3.17	112.80 ± 4.43	125.70 ± 3.55	140.40 ± 6.03
*t*	0.21	2.90	2.17	1.65
*p*	0.8321	0.0048 **	0.0333 *	0.1029

Note: * indicates significant difference (*p* < 0.05); ** indicates significant difference (*p* < 0.01).

### 3.3. Comparison of Functional Exercise Adherence (Table 9 and Figure 5E)

Functional exercise adherence scores did not differ significantly between the two groups at baseline (*p* > 0.05). After intervention, adherence scores progressively increased over time at all assessment points for both groups. Inter-group comparisons revealed significantly higher adherence scores in the intervention group at all post-intervention time points compared to the control group, with all differences being statistically significant (*p* < 0.05).

**Table 9:** Functional Exercise Compliance Score ($\bar{X}$ ± s).

Group	One Month Post-Surgery	Two Months Post-Surgery	Three Months Post-Surgery	Four Months Post-Surgery
Control Group	64.63 ± 3.09	65.63 ± 3.09	64.93 ± 3.75	61.18 ± 3.07
Trial Group	65.10 ± 3.13	67.26 ± 3.19	67.49 ± 4.16	63.10 ± 3.13
*t*	0.68	2.31	2.88	2.76
*p*	0.4965	0.0236 *	0.0052 **	0.0071 **

Note: * indicates significant difference (*p* < 0.05); ** indicates significant difference (*p* < 0.01).

### 3.4. Safety and Adverse Events

No serious adverse events, including reinjury, severe pain exacerbation, or intervention-related complications, were reported in either group during the study period. All participants completed the prescribed rehabilitation training safely under supervision.

## 4. Discussion

The present randomised controlled trial investigated the effects of an AI-based assessment and correction system rehabilitation programme on patients undergoing ACL reconstruction rehabilitation. The principal findings demonstrated that patients receiving AI-guided rehabilitation achieved significantly greater improvements in knee function, balance ability, joint range of motion, and functional exercise adherence compared with those undergoing conventional rehabilitation. These findings support the potential clinical value of integrating AI-based assessment and correction into postoperative rehabilitation programmes.

a. The intelligent assessment and training system achieves precision and personalisation in rehabilitation evaluation and training, serving as the critical driver of improved functional recovery.

Conventional rehabilitation models rely on therapists’ experience for subjective assessment and guidance, which leads to limitations such as inconsistent standards and insufficient quantification [17]. The core advantage of the intelligent system employed in the experimental group of this study lies in its utilisation of non-wearable three-dimensional motion capture and biomechanical analysis technology. This approach enables non-invasive, precise quantitative assessment of key indicators, including joint range of motion, movement symmetry, stability, and deviation angles. Through specific movement tests like squats and lunges, the system precisely identifies individualised functional impairment patterns (e.g., knee varus, hypoflexion, poor dynamic stability). Exercise prescriptions derived from this objective data are not only highly targeted but dynamically adjusted throughout the rehabilitation process. During AI-guided training, real-time visual feedback and correction ensure the quality of each movement execution, preventing the formation of incorrect compensatory patterns. This facilitates more efficient and safer restoration of neuromuscular control, joint stability, and range of motion. These mechanisms collectively account for the significantly superior Lysholm scores, IKDC scores, and ROM observed in the experimental group compared with the control group.

b. Real-time feedback and gamified design substantially enhance patient engagement and rehabilitation adherence.

Poor adherence to rehabilitation exercises poses a major challenge to functional recovery following ACL reconstruction. Previous studies have demonstrated a strong association between postoperative joint function recovery and adherence to rehabilitation exercise programmes [18,19]. However, only 30% of ACL reconstruction patients demonstrate good rehabilitation exercise adherence. Some patients underestimate the importance of rehabilitation training; inadequate exercise readily leads to restricted knee joint mobility, adversely affecting patients’ activities of daily living to varying degrees and consequently undermining their confidence in recovery [18,20]. The results of the present study show that the experimental group achieved significantly higher compliance scores than the control group, largely attributable to the design principles of the intelligent rehabilitation programme. First, the real-time feedback system—incorporating voice prompts (e.g., “knees turning in, please abduct”), graphical overlay comparisons, and movement quality ratings such as “Standard” or “Needs improvement”—provides immediate and visualised performance feedback. This satisfies patients’ cognitive need to understand training progress and movement accuracy, thereby enhancing their sense of self-efficacy. Second, gamified features, including progress tracking, achievement badges, and real-time displays of heart rate and caloric expenditure, transform repetitive exercises into an engaging and interactive experience. This effectively stimulates intrinsic motivation and encourages sustained participation. By addressing monotony, isolation, and lack of supervision, this immersive training model overcomes key limitations of traditional home-based rehabilitation. Nevertheless, both groups demonstrated reduced compliance with functional rehabilitation exercises at four months postoperatively, which may be related to improving knee function and emerging patient fatigue.

c. The intelligent solution optimises the role of rehabilitation therapists, promoting standardisation and efficiency in the rehabilitation process.

Previous research on gait recovery suggests that robot-assisted gait therapy (RAGT) may offer potential benefits for restoring walking function in patients with gait impairments. However, compared with conventional gait rehabilitation, RAGT is often characterised by high costs and uncertain clinical benefits, leaving its cost-effectiveness open to further optimisation [21,22,23]. In contrast, the intelligent rehabilitation system employed in this study does not replace rehabilitation therapists but rather elevates and refines their role. The system assumes responsibility for repetitive, standardised assessment, counting, and basic correction tasks, freeing therapists from laborious physical work. This allows therapists to focus on higher-level clinical responsibilities, including prescription review and optimisation, patient safety monitoring, and the delivery of motivational interviewing and health education. This ‘human–machine collaboration’ model not only enhances the efficiency and quality of individual rehabilitation sessions while reducing human assessment errors but also advances the standardisation and data-driven evolution of ACL postoperative rehabilitation protocols. Ultimately, this approach enhances the replicability and scientific rigour of rehabilitation programmes, paving a viable pathway for scaling evidence-based precision rehabilitation practices across broader clinical settings

d. Limitations and Future Directions.

Several limitations of this study should be acknowledged. First, the study was conducted at a single centre with a relatively short follow-up period, which may limit the generalizability of the findings and preclude assessment of long-term outcomes. Second, although functional outcomes and adherence were evaluated, biomechanical parameters and return-to-sport outcomes were not assessed.

Future multicentre studies with longer follow-up durations are warranted to validate these findings and to explore the long-term impact of AI-guided rehabilitation on reinjury risk and return-to-sport performance. Additionally, further research should investigate the cost-effectiveness and scalability of AI-based rehabilitation systems in routine clinical practice.

In conclusion, AI-based functional assessment and correction-guided rehabilitation was associated with superior postoperative recovery outcomes following ACL reconstruction compared with conventional rehabilitation. These findings provide evidence supporting the integration of AI technologies into postoperative rehabilitation programmes.

## 5. Conclusions

In this randomised controlled trial, artificial intelligence-based functional assessment and correction-guided rehabilitation was associated with superior postoperative recovery outcomes following anterior cruciate ligament reconstruction compared with conventional rehabilitation. Patients receiving AI-guided rehabilitation demonstrated greater improvements in knee function, balance ability, joint range of motion, and functional exercise adherence.

These findings suggest that integrating AI-based assessment and feedback systems into postoperative rehabilitation programmes may enhance rehabilitation efficiency and patient engagement by providing objective, individualised guidance. AI-based rehabilitation should be considered a complementary tool to clinical expertise rather than a replacement for therapist-led care.

Further multicentre studies with longer follow-up periods are warranted to confirm these findings and to evaluate the long-term clinical and functional benefits of AI-guided rehabilitation.

## Figures and Tables

**Table 1:** Comparison of basic patient characteristics between the two groups.

Group	Sample Size	Gender (Sample Size)		Age	Education (Sample Size)			BMI	Operated limb (Sample Size)		Surgery Time
		Male	Female	$\left(\overset{-}{x}\;\pm \;s\right)$	Bachelor’s Degree or Above	Associate Degree	Technical Secondary Education or Below	(kg/cm,$\overset{-}{x}\;\pm \;s$)	Left	Right	(min, $\overset{-}{x}$ ± s)
Control Group	*n* = 40	35	5	31.28 ± 7.93	22	10	8	24.87 ± 2.88	21	19	85.03 ± 24.18
Trial Group	*n* = 39	35	4	30.49 ± 10.26	15	10	13	24.78 ± 2.66	20	19	98.14 ± 38.61
Statistical quantity		*χ*^2^ = 0.10		*t* = 0.38	*χ*^2^ = 2.47			*t* = 0.14	*χ*^2^ = 0.01		*t* = 1.74
*p*		0.7537		0.7032	0.2915			0.8919	0.9137		0.0855

**Table 2:** Analysis of specific warm-up exercises and key points.

Exercise Name	Primary Target Muscle Groups	Secondary Muscle Groups	Purpose of Exercise	Key Points of the Movement
Straight-leg raise	quadriceps femoris muscle	iliopsoas muscle, core muscle group	Enhance knee joint stability and improve knee extension function.	Lie on your back with one leg bent and flat on the floor, the other leg straight with the toes pointed. Slowly raise the straight leg upwards until it reaches the height of the opposite knee. Hold for several seconds before slowly lowering it back down.
Toe stand exercise	triceps surae	plantar muscle groups, ankle joint stabilising muscle groups	Enhance ankle propulsive force, improve ankle joint stability, and utilise the calf muscle pump to promote lower limb blood circulation.	Stand upright, with hands resting on a wall for support. Slowly raise your heels to their highest point, feeling the calf muscles fully contract. Pause briefly before lowering them slowly.
Stamping both feet on the ground	tibialis anterior	extensor digitorum longus, extensor hallucis longus	Enhance ankle dorsiflexion strength to prevent shuffling gait and maintain lower leg muscle balance.	Seated position: keeping the thighs stationary, rapidly alternate between flexing the toes (dorsiflexion) and relaxing the foot, as if lightly tapping the floor with the toes.
Prone Alternating Leg Raises	luteus maximus	hamstring muscles, erector spinae	Activate and strengthen the gluteal muscles to improve pelvic stability and gait.	Prone position, abdomen engaged, pelvis stable. Extend one leg straight, using gluteal muscles to lift the leg off the ground. Pause briefly at the highest point before lowering slowly.

**Table 4:** Analysis of specific movements and key points in stretching exercises.

Movement Name	Primary Target Muscle Groups	Secondary Muscle Groups	Purpose of Exercise	Key Points of the Movement
Supine assisted stretch	hamstring muscles	triceps surae, lower back muscles	Improve hip joint mobility, prevent hamstring strains, and alleviate lower back tension.	Lie on your back with one leg straight or bent and flat on the floor, the other leg extended. Loop a towel around the sole of the foot and slowly pull with both hands, drawing the leg towards the body until a stretch is felt along the back of the thigh.
Wall-push calf stretch	triceps surae		Increase ankle dorsiflexion range of motion to prevent calf strains and alleviate plantar fascia tension.	Stand facing a wall, step forward into a lunge position with one leg, keeping the rear leg straight (to stretch the gastrocnemius) or slightly bent (to stretch the soleus). Keep the heel grounded and lean forward until a stretch is felt in the calf.
Assisted front group stretching	tibialis anterior	anterior muscle groups of the lower leg	Relieve tension and pain in the tibialis anterior muscle and improve ankle joint flexibility.	Lie on your side with the lower leg naturally flexed for stability. The physiotherapist stands behind the patient, supporting the ankle of the upper leg with one hand while gently pressing above the knee with the other. Slowly extend the leg posteriorly until a mild stretch is felt in the tibialis anterior muscle. Hold for several seconds before returning to the starting position.

## Data Availability

The datasets used and/or analysed in the current study are available from the corresponding author upon reasonable request.
